# Human hepatic stem cells transplanted into a fulminant hepatic failure Alb-TRECK/SCID mouse model exhibit liver reconstitution and drug metabolism capabilities

**DOI:** 10.1186/s13287-015-0038-9

**Published:** 2015-03-26

**Authors:** Ran-Ran Zhang, Yun-Wen Zheng, Bin Li, Tomonori Tsuchida, Yasuharu Ueno, Yun-Zhong Nie, Hideki Taniguchi

**Affiliations:** Department of Regenerative Medicine, Graduate School of Medicine, Yokohama City University, 3-9 Fuku-ura, Kanazawa-ku, Yokohama, Kanagawa 236-0004 Japan; Department of Advanced Gastroenterological Surgical Science and Technology, Faculty of Medicine, University of Tsukuba, Tsukuba, 305-8575 Japan; Oregon Stem Cell Center, Oregon Health and Science University, Portland, OR 97239 USA; Advanced Medical Research Center, Yokohama City University, 3-9 Fuku-ura, Kanazawa-ku, Yokohama, Kanagawa 236-0004 Japan

## Abstract

**Introduction:**

Chimeric mice with humanized livers were recently established by transplanting human hepatocytes. This mouse model that is repopulated with functional human hepatocytes could be a useful tool for investigating human hepatic cell biology and drug metabolism and for other preclinical applications. Successfully transplanting human hepatocytes into mice requires that recipient mice with liver failure do not reject these human cells and provide a suitable microenvironment (supportive niche) to promote human donor cell expansion and differentiation. To overcome the limitations of current mouse models, we used Alb-TRECK/SCID mice for *in vivo* human immature hepatocyte differentiation and humanized liver generation.

**Methods:**

1.5 μg/kg diphtheria toxin was administrated into 8-week-old Alb-TRECK/SCID mice, and the degree of liver damage was assessed by serum aspartate aminotransferase activity levels. Forty-eight hours later, mice livers were sampled for histological analyses, and the human donor cells were then transplanted into mice livers on the same day. Chimeric rate and survival rate after cell transplantation was evaluated. Expressions of human hepatic-related genes were detected. A human albumin enzyme-linked immunosorbent assay was performed after 50 days of transplantation. On day 60 after transplantation, drug metabolism was examined in mice.

**Results:**

Both human primary fetal liver cells and hepatic stem cells were successfully repopulated in the livers of Alb-TRECK/SCID mice that developed lethal fulminant hepatic failure after administering diphtheria toxin; the repopulation rate in some mice was nearly 100%. Compared with human primary fetal liver cells, human hepatic stem cell transplantation rescued Alb-TRECK/SCID mice with lethal fulminant hepatic failure, and human hepatic stem cell-derived humanized livers secreted more human albumin into mouse sera and also functioned as a “human liver” that could metabolize the drugs ketoprofen and debrisoquine.

**Conclusion:**

Our model of a humanized liver in Alb-TRECK/SCID mice may provide for functional applications such as drug metabolism, drug to drug interactions, and promote other *in vivo* and *in vitro* studies.

**Electronic supplementary material:**

The online version of this article (doi:10.1186/s13287-015-0038-9) contains supplementary material, which is available to authorized users.

## Introduction

Because biomedical research cannot be performed in humans, investigators commonly use mice for pharmaceutical testing [1], although these models are not always useful. Most medically used drugs are primarily metabolized in the liver. However, the same drug can be metabolized into different metabolites in mouse and human livers due to species differences. Thus, it is quite often difficult to determine whether a potential drug poses any risks during development for clinical applications [2,3].

To address this problem, “humanized” mouse livers were developed by growing human liver tissues inside mice [4-6]. These models exhibited responses to drugs similar to those of the human liver. Current mouse models used for humanized liver generation are primarily uPA+/+ (uroplasminogen activator) mice [4,7], Fah−/− (fumarylacetoacetate hydrolase) mice [6], and a recently reported TK-NOG (thymidine kinase) mouse.

However, previous reports showed that transplanted human immature cells or stem cells were less competitive as compared with human adult hepatocytes in Alb-uPAtg(+/−)Rag2(−/−) mouse livers [8-10]. Moreover, Fah−/− mice could only provide a growth advantage for differentiated hepatocytes but not for immature liver progenitor cells [11]. In our laboratory, we also failed to transplant human hepatic stem cells (HpSCs) into TK-NOG mice. Thus, no useful mouse model for the efficient engraftment of human immature liver cells currently exists.

To overcome this problem, we report here on a novel Alb-TRECK/SCID mouse model that could be efficiently repopulated with human immature hepatocytes. This transgenic mouse expresses human heparin-binding epidermal growth factor-like receptor (HB-EGF)-like receptors under the control of a liver cell-specific albumin promoter. After administering diphtheria toxin (DT), this model mouse developed fulminant hepatitis due to conditionally ablated hepatocytes, which provided space for donor cell residency and proliferation [12]. Previous studies successfully transplanted mouse hepatocytes into Alb-TRECK/SCID mice [13,14], but there have been no reports of generating a humanized liver using Alb-TRECK/SCID mice.

In this study, we generated humanized livers in Alb-TRECK/SCID mice by transplanting human primary fetal liver cells (FLCs) and HpSCs. This humanized liver provided an *in vivo* environment for universal stem cell differentiation and also an opportunity to predict the patterns of human drug metabolism and drug-to-drug interactions.

## Methods

### Acute liver injury mouse model

Alb-TRECK/SCID mice were provided by our collaborators at the Tokyo Metropolitan Institute of Medical Science. Homozygosity was confirmed by backcrossing for at least three generations. Alb-TRECK/SCID mice were housed at Yokohama City University. Animal experimental work was conducted in accordance with the Guidelines for Proper Conduct of Animal Experiments (Science Council of Japan), and all experimental procedures were approved by the institutional review board of the Animal Research Center, Yokohama City University School of Medicine (No.075).

DT (Sigma, St Louis, MO, USA; D0564-1MG) was intraperitoneally administered (1.5 μg/kg) to 8-week-old Alb-TRECK/SCID mice, and the degree of liver damage was assessed by serum aspartate aminotransferase (AST) activity levels.

### Donor cell culture

Human primary FLCs of embryonic age between weeks 14 and 18 were obtained from Cell Systems (Kirkland, WA, USA). This study was conducted with the approval of the ethics committee of Yokohama City University (Approval No. A100903011).

Human primary FLCs were cultured in Dulbecco’s modified Eagle’s medium with Ham’s F-12 nutrient mixture (1:1 mixture; Sigma, St. Louis, MO, USA) supplemented with 10% fetal bovine serum, human γ-insulin (1.0 μg/mL; Wako, Tokyo, Japan), nicotinamide (10 mM; Sigma), dexamethasone (100 nM; Sigma), and L-glutamine (2 mM; Gibco, Carlsbad, CA, USA) in dishes coated with type IV collagen (Becton Dickinson Labware). After the first 24 hours of culture, human recombinant hepatocyte growth factor (50 ng/mL; Sigma) and epidermal growth factor (10 ng/mL; Sigma) were added.

For cell passaging, culture medium was removed, cells were treated with 0.05% trypsin- ethylenediaminetetraacetic acid (Gibco) at room temperature for 5 minutes and then gently detached from the dish. Suspended cells were neutralized and washed with culture medium that contained 10% fetal bovine serum. The viability of dissociated cells was never <90% based on trypan blue exclusion.

Human HpSCs were isolated using a DakoCytomation MoFlo high-speed cell sorter (Beckman Coulter, Pasadena, CA, USA) with cell antigens that included CDCP1, CD90, and CD66 for a CDCP1^+^CD90^+^CD66^−^ population. Details for cell isolation were previously described [15]. These cells were cultured using the same procedures as for human primary FLCs.

### Liver biochemistry tests

After DT injection, blood samples were obtained from a mouse tail vein every 24 hour and centrifuged at 4,000 rpm at 4°C for 20 minutes. Serum samples were assayed for serum AST activity measured with a FUJIFILM Kit according to the manufacturer’s instructions (FUJIFILM, Tokyo, Japan). Serum AST activity levels of mice used for cell transplantation was measured at 48 hours after DT injection.

### Cell transplantation

Prior to cell transplantation, serum AST activity levels were checked, and mice with AST values between 12,000 and 16,000 IU/L were used as recipients. When cultured human primary FLCs or HpSCs reached 90% confluence, these cells were detached and adjusted to a final concentration of 1 × 10^6^ viable cells per 50 μL culture medium. Human primary FLCs or HpSCs (1 × 10^6^) were transplanted into the spleens of Alb-TRECK/SCID mice. Mice in the sham group received 50 μL sterile saline.

### BrdU injection

At 46 hours after DT was injected into Alb-TRECK/SCID mice, BrdU (50 mg/kg) was administered intraperitoneally to five mice in each group, and mice were sacrificed 2 hours later. Liver sections were prepared, fixed with 4% paraformaldehyde (PFA) and washed with 0.05% Tween 20 in phosphate-buffered saline (PBS). Sections were then treated with 2 N hydrochloric acid and neutralized with 0.1 M sodium tetraborate (pH 8.5). The sections were then stained with an anti-BrdU antibody (BD Pharmingen, San Jose, CA, USA), and Alexa Fluor®488 goat anti-mouse IgG1 (Invitrogen, Carlsbad, CA, USA) was used as a secondary antibody for visualization. Nuclei were stained with 4′,6-diamidino-2-phenylindole (DAPI), and sections were mounted with Apathy’s Mounting Media (Wako Pure Chemical Industries, Osaka, Japan).

### Histology and immunocytochemistry

Liver tissues were fixed with 10% neutral formalin for 2 days and washed with PBS for 1 day. After dehydration with ethanol and xylene, tissues were embedded in paraffin and serial sections were prepared (4 μm thick). These samples were stained with hematoxylin and eosin.

For double or triple immunohistochemical staining, liver tissues were frozen in optimum cutting temperature compound (Sakura, Tokyo, Japan), liver sections (5 um thick) were prepared and fixed in acetone:methanol (1:1) for 30 minutes, and then blocked with 10% normal goat serum for 60 minutes. Sections were then incubated with primary antibodies (1:200), including mouse anti-human albumin mAb (Sigma), mouse anti-human CK19 mAb (Dako, Tokyo, Japan), guinea pig anti-human CK8/18 (Progen, Heidelberg, Germany), mouse anti-human nuclei (Millipore, Billerica, MA, USA), and mouse anti-Ki67 (Dako) at 4°C overnight. Sections were washed with PBS and then incubated with appropriate Alexa-488, -555, or -647-conjugated secondary antibodies (1:500; Invitrogen) at room temperature for 60 minutes. Cells were counterstained with DAPI and sections were mounted with Apathy’s Mounting Media (Wako Pure Chemical Industries). Images were acquired using a Zeiss AxioImager and microscope (Carl Zeiss, Jena, Germany).

### Real-time PCR

Total RNAs from humanized liver tissue and human primary FLCs and HpSCs and human adult hepatocytes were extracted using Isogen reagent (Nippon Gene, Toyama, Japan). cDNA was synthesized with a High Capacity cDNA Reverse Transcription Kit (Applied Biosystems, Foster, CA, USA). Quantitative PCR was performed according to the manufacturer’s protocol using TaqMan Gene Expression Assays (Applied Biosystems) and data were analyzed with an ABI PRISM® 7900HT Sequence Detection System (Applied Biosystems). Probes used were ALB (Hs00609411_m1), AFP (Hs01040607_m1), CYP3A4 (Hs01546612_m1), CYP2C9 (Hs00426397_m1), CYP2C19 (Hs00426380_m1), and hACTB (4326315E). TaqMan Gene Expression Assay IDs (Applied Biosystems) are shown in parentheses after the gene names.

### Microarray analysis

Total RNAs were extracted from human primary FLCs, HpSCs, and Alb-TRECK/SCID mouse livers that received cell transplants separately for three independent experiments using an RNeasy Mini Kit (Qiagen, Venlo, Netherlands). RNA samples were individually hybridized to a pool of two commercial normal ovary RNA on a Whole Human Genome Agilent 4 × 44 K v2 Oligonucleotide Microarray (Agilent Technologies, Santa Clara, CA, USA), according to the manufacturer’s instructions. For cross-species comparisons of expression profiles, total expression data at the gene level were cross-referenced to other species using the HomoloGene IDs in the Mouse Genome Informatics curated data set of human–mouse orthology with Phenotype Annotations [16]. To generate a heat map, we used a hierarchical clustering method with Euclidean distances for complete linkage on GeneSpring11.5.1. to analyze 83 and 38 selected gene expression profiles. The raw data of the microarray analysis have been deposited in the Gene Expression Omnibus database (GSE62933).

### Albumin assay

Blood samples (20 μl) were collected periodically from mouse tail veins and centrifuged at 4,000 rpm at 4°C for 20 minutes. Serum samples were assayed for human albumin using a human albumin enzyme-linked immunosorbent assay (ELISA) quantitation kit (Bethyl Laboratories Inc., Montgomery, TX, USA), according to the manufacturer’s instructions. After 6 minutes, reactions were stopped by adding 2 M sulfuric acid, and absorbance was read at 450 nm using a Multimode Detector DTX 880 (Beckman Coulter, Pasadena, CA, USA).

### Drug metabolite detection

At about 7 to 8 weeks, mice without and with cell transplantation were intravenously administrated ketoprofen (15 mg/kg), and urine samples were collected from 0 to 2 hours in 0.5 M acetate buffer (pH 5.0). For debrisoquine (DEB) metabolic testing, mice were administered DEB (2 mg∕kg) by oral gavage, and then blood samples were obtained from tail veins at 0, 0.5, 1, 2, 4, and 8 hours with heparin-Na added. Plasma was separated from blood by centrifugation. Metabolites were quantified using an LC-20A Series liquid chromatography-tandem mass spectrometer (Shimadzu, Kyoto, Japan) with an Intersil ODS-3 column (GL Science Co., Tokyo, Japan). The details were previously described [17].

### Statistical analysis

Results for two groups were statistically compared using the Mann–Whitney U-test and results for more than two groups were compared by one-way analysis of variance and Bonferroni multiple comparisons tests. A log-rank (Mantel–Cox) test and the Kaplan–Meier method were used to assess post-transplantation survival. A *P*-value of <0.05 was considered significant. Statistical analysis was performed using Graphpad Prism software (San Diego, CA, USA).

## Results

### Diphtheria toxin induces lethal fulminant hepatic failure in Alb-TRECK/SCID mice

Alb-TRECK/SCID mice hepatocytes harbor the gene for the human DT receptor, HB-EGF, under the control of an albumin promoter, and exhibit cytotoxic effects after DT administration. To evaluate the effects of DT injection on liver injury, we injected DT doses of 0.5, 1, 1.5, 2, and 5 μg/kg into groups of 8-week-old Alb-TRECK/SCID mice (five per group) and measured serum AST activity levels every 24 hours up to 96 hours after administration of DT.

**Figure 1:**
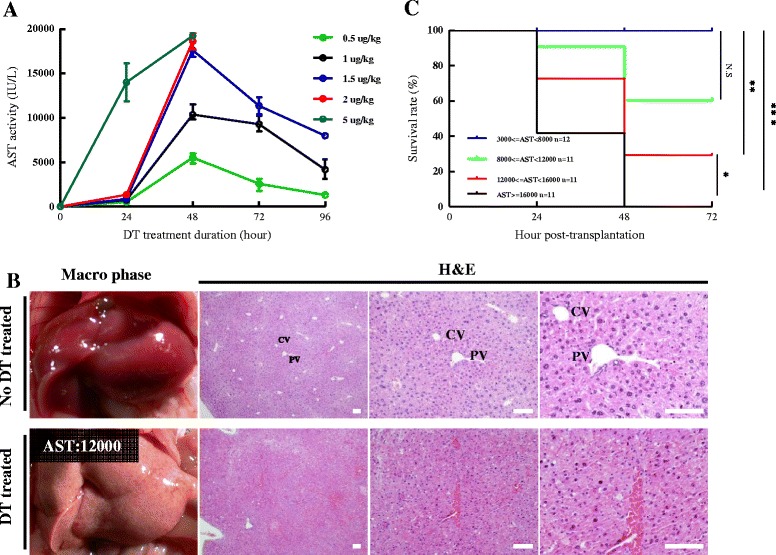
**Alb-TRECK/SCID mice develop fulminant hepatic failure after diphtheria toxin injection. (A)** Serum aspartate transaminase (AST) activity levels over time after diphtheria toxin (DT) injection during the first 96 hours. DT doses of 0.5, 1, 1.5, 2, and 5 μg/kg were injected into 8 week-old Alb-TRECK/SCID mice, and serum AST activity was determined every 24 hours. Results are means ± standard error of the mean (n = 5/group). **(B)** Macroscopic views (left panels) and histology (hematoxylin and eosin (H&E) staining, right three panels) of mouse livers without and with administration of DT after 48 hours. DT dose: 1.5 μg/kg, serum AST activity of normal liver: 30 IU/L; serum AST activity of DT-treated liver: 12,000 IU/L. CV, central vein; PV, portal vein. Scale bars = 100 μm. **(C)** Kaplan–Meier survival curves of Alb-TRECK/SCID mice with different AST activities within 3 days after DT injection (numbers of mice in each group are indicated in the figure). *P < 0.05, **P < 0.01, ***P < 0.001. NS, not significant.

Serum AST activity reached a peak at 48 hours after DT injection and then returned toward basal levels by 96 hours (Figure 1A). This indicated that acute liver failure might have occurred and that the most severe liver damage might have been induced 48 hours after the administration of DT. All mice were dead within 48 hours after receiving 2 and 5 μg/kg of DT, and three mice were dead by 72 hours while two mice survived after administration of 1.5 μg/kg DT. All mice survived after administration of 0.5 and 1 μg/kg DT. Thus, we defined 1.5 μg/kg DT for 48 hours as a “sub-lethal dose” that could induce fulminant hepatic failure.

Next, we histologically assessed pathological changes in the liver at 48 hours after administration of DT in 8-week-old mice with different serum AST activities. This showed that, as compared with normal mice which were not administered DT, after administration of DT there was a disorganized hepatic architecture showing a correction for congestion with the increased serum AST activity, and hepatocytes also had multiple, deeply stained acidophilic cytoplasmic inclusions along with dark nuclei (most probably apoptotic hepatocytes), while other hepatocytes appeared either preserved or exhibited a vacuolated cytoplasm with dark nuclei, along with little or no portal vein inflammation (Figure 1B and Additional file 1: Figure S1).

Furthermore, after DT injection, all of the mice with AST values of <8,000 IU/L had survived, the survival rate of mice with AST values between 8,000 and 12,000 IU/L declined to about 60%, and it further declined to 25% when the AST values were between 12,000 and 16,000 IU/L. All of the mice with serum AST activity levels of >16,000 IU/L were dead within 48 hours (Figure 1C). These results were in agreement with those of a previous study that showed that Alb-TRECK/SCID mice were an ideal lethal fulminant hepatic failure model generated by only a single DT injection [12,14].

### Mouse hepatocyte proliferation is induced in response to fulminant hepatic liver failure

**Figure 2:**
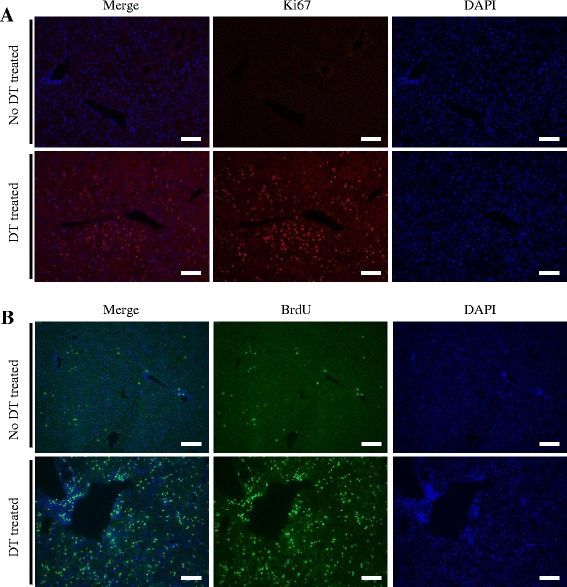
**Extensive mouse hepatocyte proliferation after administration of diphtheria toxin.** Immunofluorescent staining for cell proliferation markers Ki67 **(A)** and BrdU **(B)** in liver tissues from 8-week-old Alb-TRECK/SCID mice without and with diphtheria toxin (DT) treatment. DT-treated mouse liver with serum aspartate aminotransferase activity of 12,000 IU/L was sampled at 48 hours. At 2 hours before sampling, BrdU (50 mg/kg) was administered intraperitoneally. Experiments were performed with five mice/group, and representative images are shown. Nuclei were counterstained with 4′,6-diamidino-2-phenylindole (DAPI). Scale bars = 100 μm.

To assess *in vivo* hepatocyte proliferation after the administration of DT, we performed an immunohistochemical analysis using cell cycle markers for total cell cycle activity (Ki-67) and S-phase progression (BrdU incorporation, Cyclin A) in the livers of both normal mice and mice with fulminant hepatic liver failure. This showed that there was a higher degree of Ki67-positive expression (Figure 2A, lower panels) and BrdU incorporation (Figure 2B, lower panels) in livers at 48 hours after the administration of DT. In contrast, no positive Ki67 (Figure 2A, upper panels) and only a few BrdU-positive cells (Figure 2B, upper panels) were detected in normal mouse livers. These results showed that mouse liver regeneration was occurring after DT injection.

### Alb-TRECK/SCID mice with lethal fulminant hepatic failure are rescued by human hepatic stem cell transplantation

**Figure 3:**
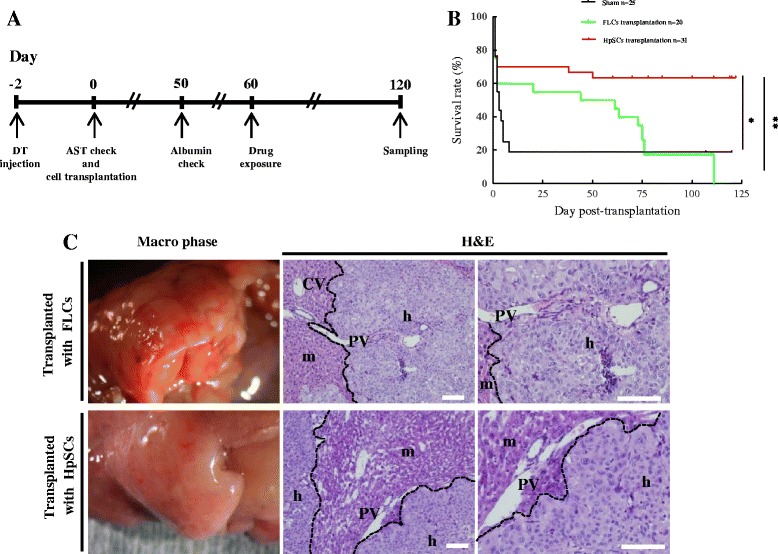
**Transplanting human primary fetal liver cells and human hepatic stem cells into Alb-TRECK/SCID mice with fulminant hepatic failure. (A)** Experimental protocols. Forty-eight hours after the intraperitoneal injection of diphtheria toxin (DT), mouse serum was collected for the aspartate aminotransferase (AST) assay, and mice livers (n = 5) were sampled for histological analyses. The human donor cells were then transplanted into mice livers (n = 54/group) on the same day. A human albumin enzyme-linked immunosorbent assay was performed after 50 days of transplantation (n = 6/group). On day 60 after transplantation, drug metabolism was examined in mice (n = 4 or more/group), and over 20 mice per group were used for the survival tracing. Mice that survived for more than 120 days were sacrificed for liver tissue sampling. **(B)** Kaplan–Meier survival curves of Alb-TRECK/SCID after transplantation with human primary fetal liver cells (FLCs) and human hepatic stem cells (HpSCs). (Numbers of transplanted mice in each group are indicated in the figure.) *P < 0.05, **P < 0.01. **(C)** Macroscopic views (left panels) and histology (hematoxylin and eosin (H&E) staining, right two panels) of humanized livers with human primary FLCs (upper panel) and human HpSCs (lower panel) at 6 weeks after transplantation. CV, central vein; PV, portal vein; m, mouse liver region; h, human donor cell-derived human region. Scale bars = 100 μm.

Human immature hepatocytes, including human primary FLCs and HpSCs, could be used for long-term *in vitro* culture. Human HpSCs exhibited uniform cell morphology, with more ALB and fewer CK19-positive cells as compared with human primary FLCs (Additional file 1: Figure S2). Eight-week-old mice received DT injections (1.5 μg/kg) 2 days before cell transplantation and were checked for serum AST activity. Mice with AST activity levels between 12,000 and 16,000 IU/L were used as recipients and were transplanted with 1 × 10^6^ cells, either human primary FLCs or HpSCs as described in the experimental protocol (Figure 3A).

More than 60% of human HpSCs transplanted mice survived for more than 120 days, whereas all of the human primary FLCs transplanted mice were dead within 110 days (Figure 3B). However, human primary FLCs rescued mice in terms of survival at 7 days after transplantation, as >80% of the sham group mice with saline transplantation were dead within 7 days. Also, mice with human primary FLCs exhibited a pronounced body weight loss within 3 days after cell transplantation, whereas mice that received human HpSCs showed a gradual body weight increase similar to that of the sham group (Additional file 1: Figure S3).

Whole mouse livers at 6 weeks after cell transplantation were grossly examined under a microscope (Figure 3C, left panels) and by histological examination, which showed that both human primary FLCs and HpSCs had reconstituted the liver structure by replacing original mouse hepatocytes (Figure 3C, middle and right panels). Macroscopically, at 4 days after human HpSC transplantation, we detected small human hepatic clusters that were uniformly distributed around the liver and had proliferated into large clusters at 45 days (Additional file 1: Figure S4A). Thus, these whole mouse livers that had been replaced with human hepatocytes were designated “humanized livers” (Additional file 1: Figure S4B). These results showed that mice with lethal fulminant hepatic failure that underwent human primary FLC transplantation survived over a short term, whereas for long-term survival transplanted human HpSCs might have been functional. Both human primary FLC- and HpSC-derived humanized livers were healthy and exhibited liver structures similar to normal mouse livers.

### Proliferative human hepatic stem cells successfully differentiate in humanized livers of Alb-TRECK/SCID mice

**Figure 4:**
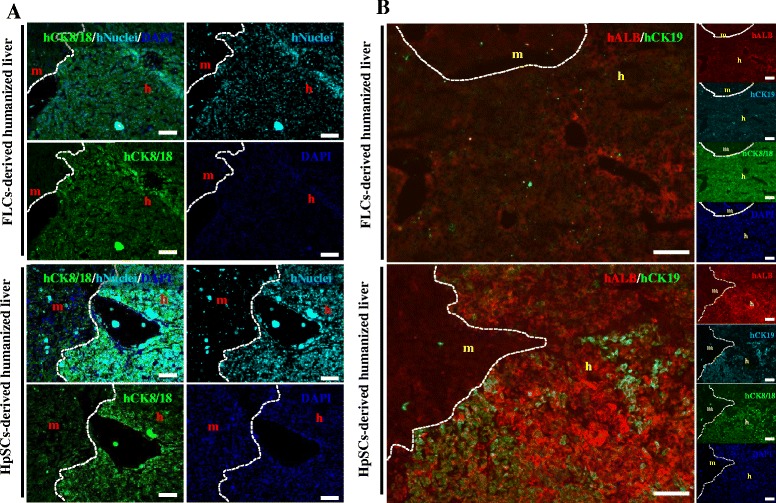
**Characterization of human primary fetal liver cell- and human hepatic stem cell-derived human hepatocytes in Alb-TRECK/SCID mice. (A)** Immunohistochemistry to distinguish between human hepatocytes stained with anti-human CK8/18 (green) antigen and anti-human nuclear antigen (aqua blue) in human primary fetal liver cell- (FLC; upper panels) and human hepatic stem cell- (HpSC; lower panels) derived humanized livers at 6 weeks after transplantation. **(B)** Immunohistochemistry analyses for human albumin, human CK19, and human CK8/18 expression in human primary FLC- (upper panels) and HpSC- (lower panels) derived livers at 6 weeks after transplantation. White dashed line: mouse liver region distinguished from human liver region. Nuclei were counterstained with 4′,6-diamidino-2-phenylindole (DAPI, blue). Scale bars = 100 μm. m, mouse liver region; h, human donor cell-derived human region.

Prior to testing whether the transplanted human immature hepatocytes in mice had the potential for differentiation and be functional *in vivo*, we firstly confirmed human hepatocytes existed in mouse livers by immunohistochemical analysis at about 6 weeks after transplanting human primary FLCs (Figure 4A, left panels) or HpSCs (Figure 4B, left panels). Liver sections from these mice that were specifically positively co-stained with human nuclei and human cytokeratin 8/18 (CK8/18) antibodies were donor cell-derived human hepatocytes, while the original liver regions in mice were negative for these markers.

To assess the degree of cell differentiation *in vivo*, we immunohistochemically assessed for human albumin (ALB) and human cytokeratin 19 (CK19) expressions. This showed that human primary FLC-derived liver sections that were positively stained with human CK8/18 were human ALB and CK19 negative (Figure 4A, right panels), whereas human HpSC-derived human hepatocytes in mouse livers were well differentiated and with upregulated human ALB expression. Human ALB-positive hepatocytes that were CK19 negative resembled functional hepatocytes, while cells that positively co-stained with human ALB and CK19 exhibited a bipotential capability with differentiation into hepatocytes and cholangiocytes (Figure 4B, right panels).

A large-scale scan method to analyze the entirety of human HpSC-derived humanized liver lobes showed that multiple round and colony-like clusters were distributed around the liver lobes with clear human nuclei, and CK8/18 and ALB expression (Additional file 1: Figure S5), indicating that the colony-forming capability of human HpSCs were maintained in humanized livers.

These results showed that, compared with human primary FLCs, human HpSCs had a high potential for differentiation into functional hepatocytes *in vivo* in response to the rescue of damaged mouse liver functions.

### Characterization of human drug metabolism gene expression in Alb-TRECK/SCID mouse with humanized livers

**Figure 5:**
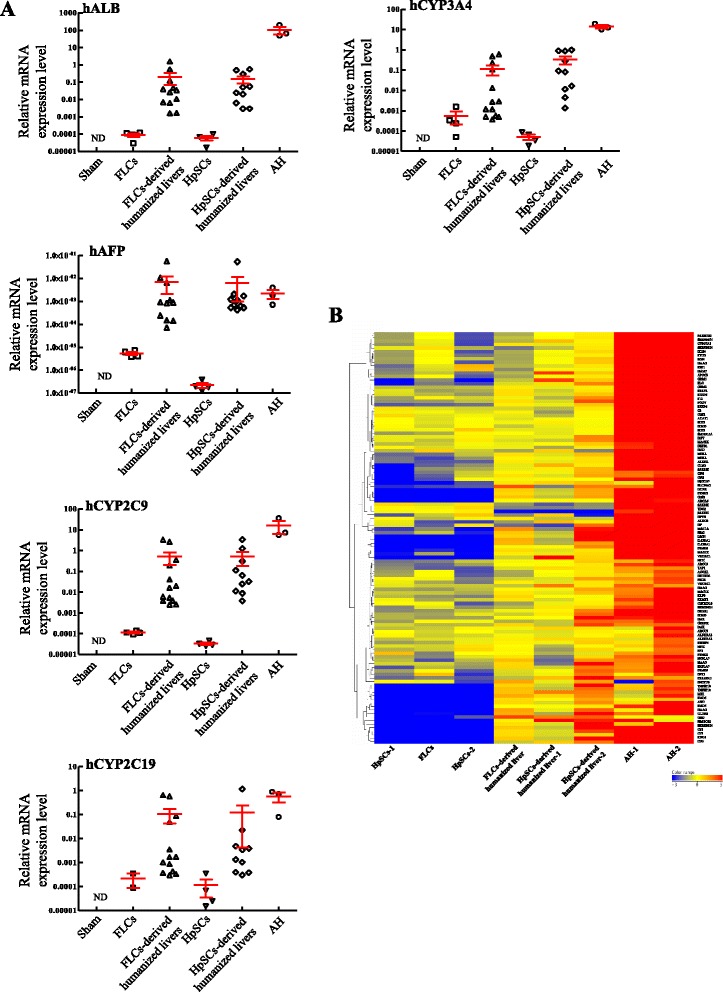
**Expressions of human hepatocyte-related genes in human primary fetal liver cell- and human hepatic stem cell-derived humanized livers. (A)** Results of quantitative PCR for the expression of functional hepatocyte markers, including hALB, hAFP, hCYP3A4, hCYP2C9, and hCYP2C19, in samples taken before and about 8 weeks after human primary fetal liver cell (FLC) and human hepatic stem cell (HpSC) transplantation. Results are mean ± standard error of the mean (n ≥ 4 mice/group). **(B)** Heat maps for 83 human-specific drug metabolism genes and transcription factors, shown separately for independent experiments to analyze human primary FLCs and HpSCs before and at 8 weeks after cell transplantation. Human adult hepatocytes were used as a positive control. AH, adult hepatocyte; ND, not detectable; Sham, mice transplanted with saline.

We also evaluated human drug metabolism-related gene expression by quantitative PCR and microarray analysis to assess whether human primary FLC- and HpSC-derived humanized livers could be used for human drug metabolism studies. At 8 weeks after transplantation, we observed very high gene expression levels associated with the human hepatic functional markers ALB, AFP, and cytochrome P450, including CYP3A4, 2C9, and 2C19, which collectively metabolize over 80% of clinical drugs (Figure 5A). Almost none of the probes on the human gene expression array had cross-hybridized with murine mRNA.

To comprehensively assess for genes associated with drug metabolizing enzymes, we performed microarray analysis for 83 previously reported human drug metabolism-related genes (Figure 5B) and 38 mature hepatocyte-specific genes (Additional file 1: Figure S6A) whose expressions were robustly increased in humanized livers. We chose the 83 genes because their expression increased continuously during both murine and human liver development [18], and the subset of 38 genes was used to identify differentiated hepatic characteristics [19]. Three pairwise comparisons selectively displayed genes with a twofold expression change (increase or decrease) in humanized livers derived from human HpSCs, human HpSCs and human adult hepatocytes, and showed that humanized livers shared 1,049 genes with human adult hepatocytes, which included liver-specific genes, ALB, AFP and ABCC6, and genes for drug metabolizing enzymes, CYP2C9, 2C19 and 2D6 (Additional file 1: Figure S6B and Additional file 2). We also found that 27 of 53 phase I, 86 of 99 phase II, and 35 of 51 phase III genes could be detected in humanized livers derived from human HpSCs, similar to that of human adult hepatocytes (Additional file 1: Figure S6C and Additional file 3). These results indicated that relevant functional human drug metabolizing enzymes were expressed in humanized livers derived from human HpSCs, which could be useful for preclinical drug development.

### Functional characterization of humanized livers in Alb-TRECK/SCID mice

**Figure 6:**
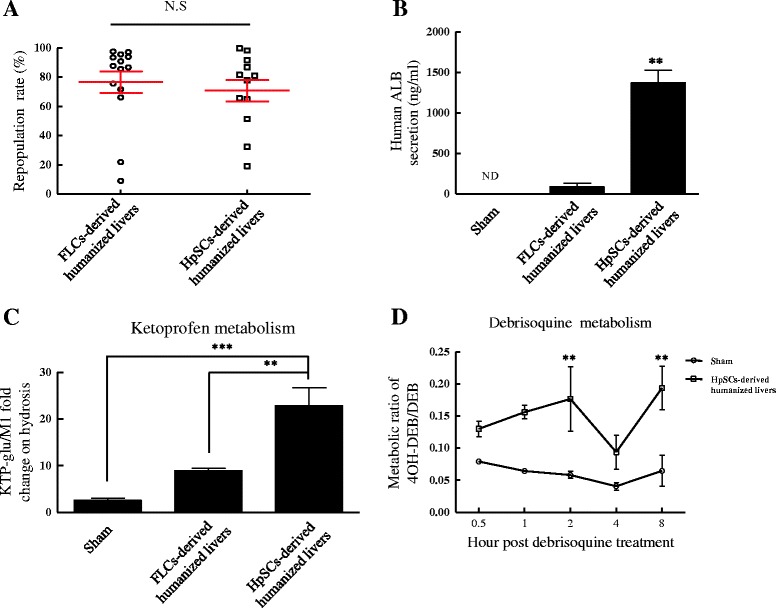
**Functional assessments of humanized livers in Alb-TRECK/SCID mice. (A)** Repopulation rates of human primary fetal liver cell (FLC)- and human hepatic stem cell (HpSC)-derived human hepatocytes in Alb-TRECK/SCID mice at about 8 weeks after transplantation. Results are means ± standard error of the mean (n = 12 and 14, respectively). **(B)** Serum human albumin (ALB) levels in mice with humanized livers derived from human primary FLCs and HpSCs at about 7 to 8 weeks after transplantation. Results are means ± standard error of the mean (n = 6/group). **(C)** Human-specific ketoprofen (KTP; CYP2C9) drug biotransformation in humanized Alb-TRECK/SCID mice at about 8 weeks after transplantation. **(D)** Analysis of human-specific CYP2D6-mediated debrisoquine (DEB) metabolism in human HpSC-derived humanized Alb-TRECK/SCID mice by metabolic ratios of the metabolite 4-hyroxydebrisoqune (4OH-DEB) to DEB. Results are means ± standard error of the mean (n ≥ 4/group). **P < 0.01, ***P < 0.001. ND, not detectable; NS, not significant; Sham, mice transplanted with saline.

At about 8 weeks after transplantation, the level of liver repopulation with human donor cells and human ALB concentrations in mouse sera were determined. The average liver repopulation rate for human primary FLC- and HpSC-derived humanized livers were 76% and 71%, respectively; no significant difference was observed. Several humanized livers reached liver repopulation levels of about 100%, which indicated that almost the entire mouse liver had been reconstituted with human hepatocytes (Figure 6A). Humanized livers derived from human HpSCs resulted in more human ALB secretion than those from human primary FLCs, and no human ALB could be detected in mice after saline transplantation (Figure 6B). The drug metabolism profiles based on gene expression and human ALB secretion patterns suggested the potential of humanized livers derived from human HpSCs for early identification of major drug metabolites *in vivo*.

We administered ketoprofen (KTP) [20] and DEB [21], which are known to be metabolized differently by mice and humans, into sham-treated mice and mice transplanted with human hepatocytes. KTP, a CYP2C9 substrate, is primarily metabolized to KTP-glucuronide (KTP-glu) by humans and metabolized to hydroxyl-KTP in mice. DEB is a prototypical CYP2D6 substrate that is converted to its 4OH metabolite (4OH DEB) by CYP2D6. The fold-change of KTP-glu/M1 was significantly greater in human HpSC-derived humanized livers than in human primary FLC-derived humanized livers and sham-treated mice (Figure 6C). For DEB, a higher metabolic ratio of 4OH-DEB/DEB was detected in human HpSC-derived humanized livers (Figure 6D). These results showed that human HpSC-derived humanized livers would be advantageous for improving the quality of human drug metabolism and for preclinical studies.

## Discussion

Recent studies have shown that mouse livers could be repopulated with human hepatocytes, including adult hepatocytes and proliferative HpSCs [22]. These were used as a preclinical experimental model for drug metabolism testing [23] and drug discovery and development [24], and they also provided an *in vivo* environment for cell maturation and differentiation [25]. The major aim of the present study was to generate a novel acute liver disease mouse model that provided for human immature hepatocyte proliferation, maturation, and differentiation in order to acquire drug metabolism activities.

It was clear that this novel Alb-TRECK/SCID mouse model developed lethal fulminant hepatic failure using one dose of DT, which provided a platform for studying the basic biology of liver regeneration after injury and liver disease development. As is known, even human immature hepatocytes can proliferate extensively *in vitro*, although they lose drug metabolism functions which limits their preclinical applications [26]. After transplanting human immature hepatocytes, including human primary FLCs and human HpSCs, into Alb-TRECK/SCID mice with lethal fulminant hepatic failure, we found histological and immunohistochemical evidence that both human primary FLCs and HpSCs could expand and reconstitute the damaged mouse liver structures; the repopulation rate in some mice was nearly 100%.

However, as compared with human primary FLCs, human HpSC transplantation promoted mouse survival and resulted in more human ALB secreted into mouse sera. These cells also exhibited maturation and differentiation *in vivo* with human drug metabolism activities, which indicated that humanized livers in mice derived from human HpSCs were similar to a mature, functional “human organ” and have potential applications in drug development. This further confirmed that Alb-TRECK/SCID mice were an ideal model for humanized liver generation.

Despite the prospects and advantages of Alb-TRECK/SCID mice as an ideal model for human HpSC-derived humanized liver generation, there was one unique disadvantage as well. We transplanted human adult hepatocyte into three Alb-TRECK/SCID mice with one DT dose treatment and failed to get the humanized livers, possibly because human adult hepatocytes lacked the capability to proliferate. Additional DT doses cannot be administered after human adult hepatocyte transplantation. Since the DT receptor (HB-EGF) in Alb-TRECK/SCID mice hepatocytes is under the control of the albumin promoter, additional DT treatment will destroy transplanted human adult hepatocytes in the mouse liver.

Thus, Alb-TRECK/SCID mice can sustain acute liver injuries with only one DT dose injection and are easily bred recipients for human immature hepatocyte transplantation. They are an acceptable model for human HpSC transplantation as they provide a beneficial environment that allows for the differentiation into mature hepatocytes. These mice are certainly an interesting model that has tremendous potential applications, not only for *ex vivo* expansion of human hepatocytes, but also to test candidate therapeutic drugs for liver toxicity and metabolism as well as for drug screens.

## Conclusions

Overall, we have shown that Alb-TRECK/SCID mice are an ideal model for induced lethal fulminant hepatic failure that could be used to study hepatocyte regeneration and liver disease development and facilitate *in vivo* human immature hepatocyte differentiation; it also has the potential for human drug metabolism testing. After human immature hepatocytes, including human primary FLCs and HpSCs, were transplanted into Alb-TRECK/SCID mice administered DT, damaged mouse livers were reconstituted with high liver repopulation rates. Furthermore, human HpSC transplantation-derived humanized livers exhibited higher human liver functions, including human ALB secretion and drug metabolism capabilities. Thus, our model of humanized livers in Alb-TRECK/SCID mice allows for the use of functional applications, such as examinations of drug metabolism, drug–drug interactions, and hepatic virus transfection, and the promotion of human HpSC-related studies, such as *in vivo* evaluation of stem cell differentiation and development processes.

## Additional files

Additional file 1:
**Supplemental information. This file contains Figure S1-S6.**
Additional file 2:
**This file contains a list of global gene expression profiles in each of the comparisons, referred to as Figure S6B.**
Additional file 3:
**This file contains a list of drug metabolism related phase I, phase II and phase III genes detected in human HpSCs, human HpSC-derived humanized liver and human adult hepatocytes, referred to as Figure S6C.**

## Abbreviations

ALB: albumin
AST: aspartate aminotransferase
BrdU: 5-bromo-2'-deoxyuridine
CK19: cytokeratin 19
DAPI: 4′,6-diamidino-2-phenylindole
DEB: debrisoquine
DMEM: Dulbecco’s modified Eagle’s medium
DT: diphtheria toxin
ELISA: enzyme-linked immunosorbent assay
FLC: fetal liver cell
HB-EGF: heparin-binding epidermal growth factor
HpSC: hepatic stem cell
KTP: ketoprofen
KTP-glu: KTP-glucuronide
PBS: phosphate-buffered saline
SCID: severe combined immunodeficiency
TRECK: toxin receptor mediated cell knockout
