# Supplementary material for: Human hepatic stem cells transplanted into a fulminant hepatic failure Alb-TRECK/SCID mouse model exhibit liver reconstitution and drug metabolism capabilities
Source: Stem Cell Res Ther. 2015 Mar 26;6(1):49. doi: 10.1186/s13287-015-0038-9 (PMC4414454; doi:10.1186/s13287-015-0038-9)
Supplement: Additional file 1: — Supplemental information. This file contains Figure S1-S6. [file 13287_2015_38_MOESM1_ESM.pdf]

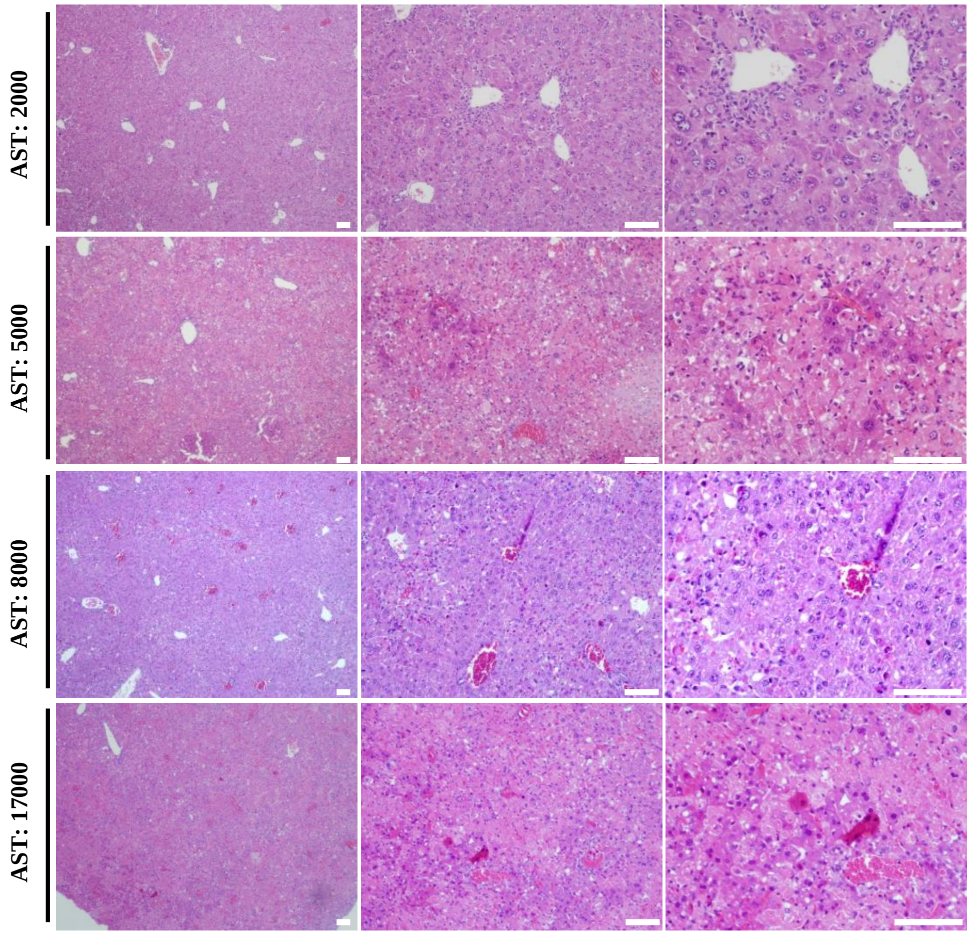

Figure S1. Histological analysis for the relationship between serum aspartate transaminase activity and the degree of liver damage.

At 48 hours after diphtheria toxin injection, serum aspartate transaminase (AST) activity was determined and Alb-TRECK/SCID mouse livers were sampled and stained with hematoxylin and eosin. Scale bars = 100 μm.

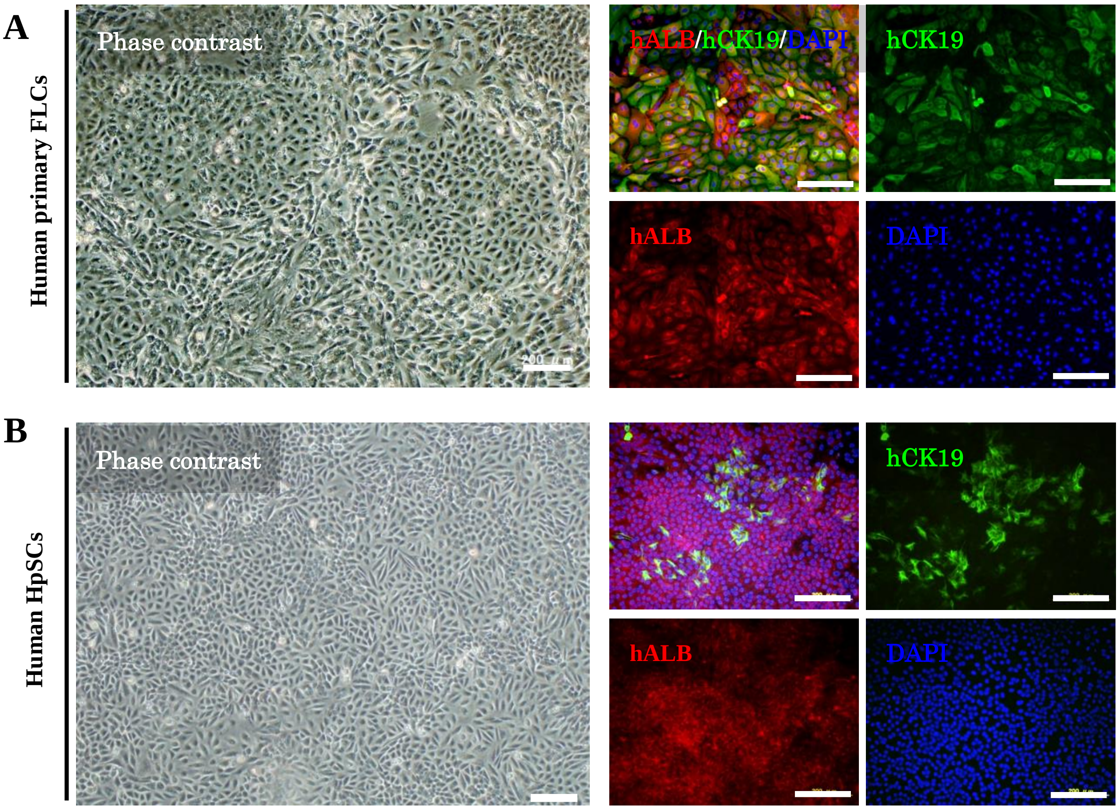

Figure S2. Characteristics of human primary fetal liver cells and human hepatic stem cells in vitro.

Morphologies (left panels) and immunocytochemistry results (right two panels) of human primary fetal liver cells (FLCs; A) and human hepatic stem cells (HpSCs; B) stained for human albumin (red) and CK19 (green). Cells were passaged 8 times. Nuclei were counterstained with 4′,6-diamidino-2-phenylindole (blue). Scale bars = 200 µm.

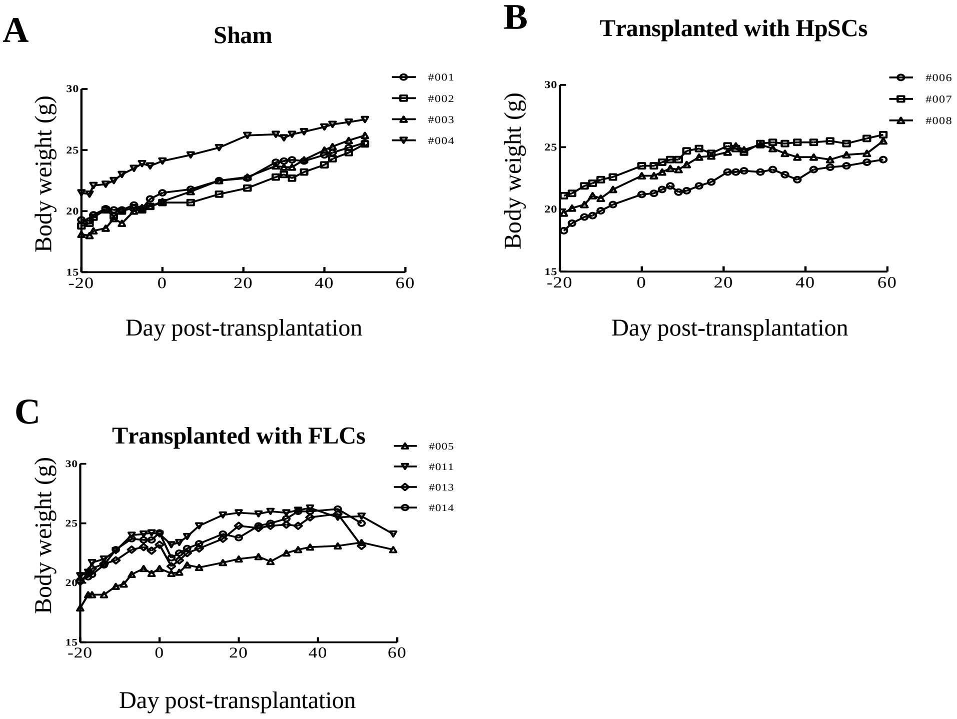

Figure S3. Body weight changes for Alb-TRECK/SCID mice after human primary fetal liver cells and human hepatic stem cells transplantation.

A-C. Mouse body weights were monitored for 20 days before and for 60 days after cell transplantation. Day 0: time point for cell transplantation. #001-#014: IDs of transplanted mice. Sham: mice transplanted with saline.

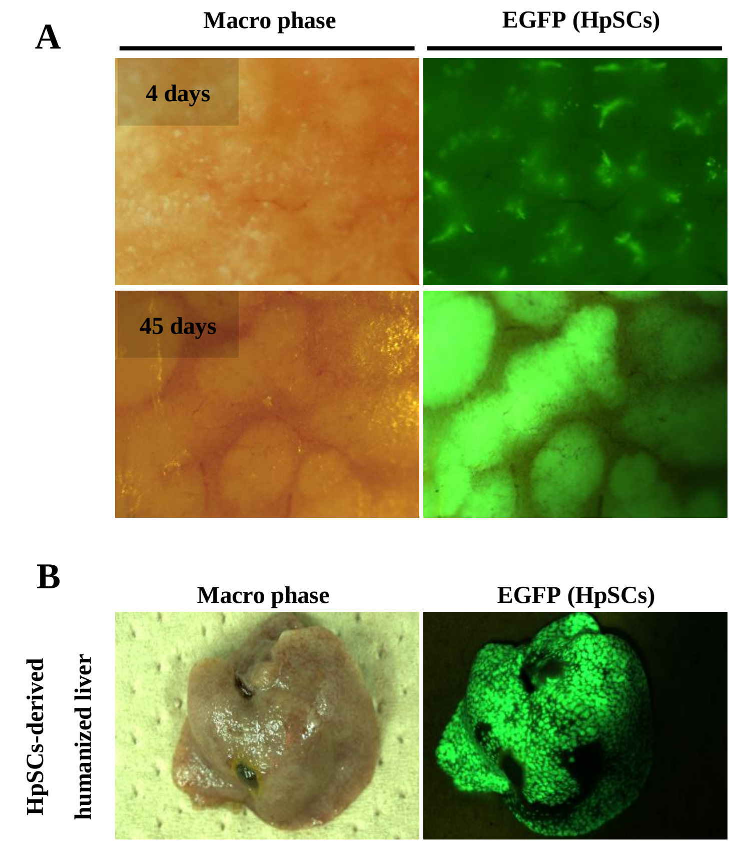

Figure S4. Macroscopic analysis of humanized livers derived from human hepatic stem cells labeled with green fluorescent protein.

A. High-magnification images of liver structure after transplantation with human hepatic stem cells (HpSCs) for 4 days and 45 days. Human hepatic stem cells (HpSCs) were labeled with green fluorescent protein (EGFP) using lentivirus. B. Macroscopic image of a whole humanized liver at 45 days after transplantation with human EGFP-HpSCs. EGFP positive cells in right panels are human hepatic stem cells (HpSCs) derived cells.

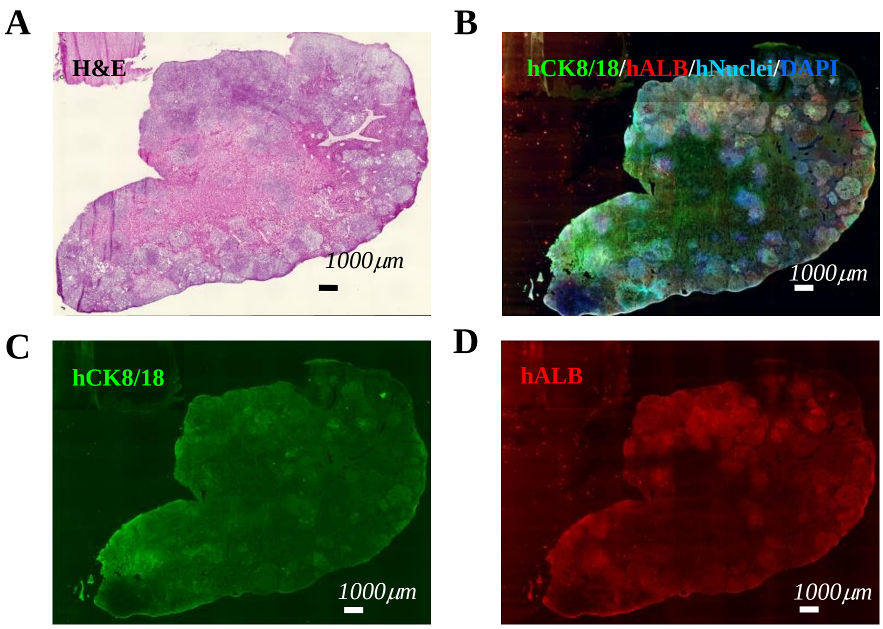

Figure S5. Human hepatic stem cell-derived multiple clusters in Alb-TRECK/SCID mouse with humanized livers.

Multiple large clusters derived from human hepatic stem cells in liver lobes at 6 weeks after transplantation were identified by hematoxylin and eosin staining (A) and immunohistochemistry analysis (B-D) for human CK8/18 (green), human nuclei (aqua blue), and human albumin (red). Nuclei were counterstained with 4′,6-diamidino-2-phenylindole (blue). Scale bars = 1000 μm.

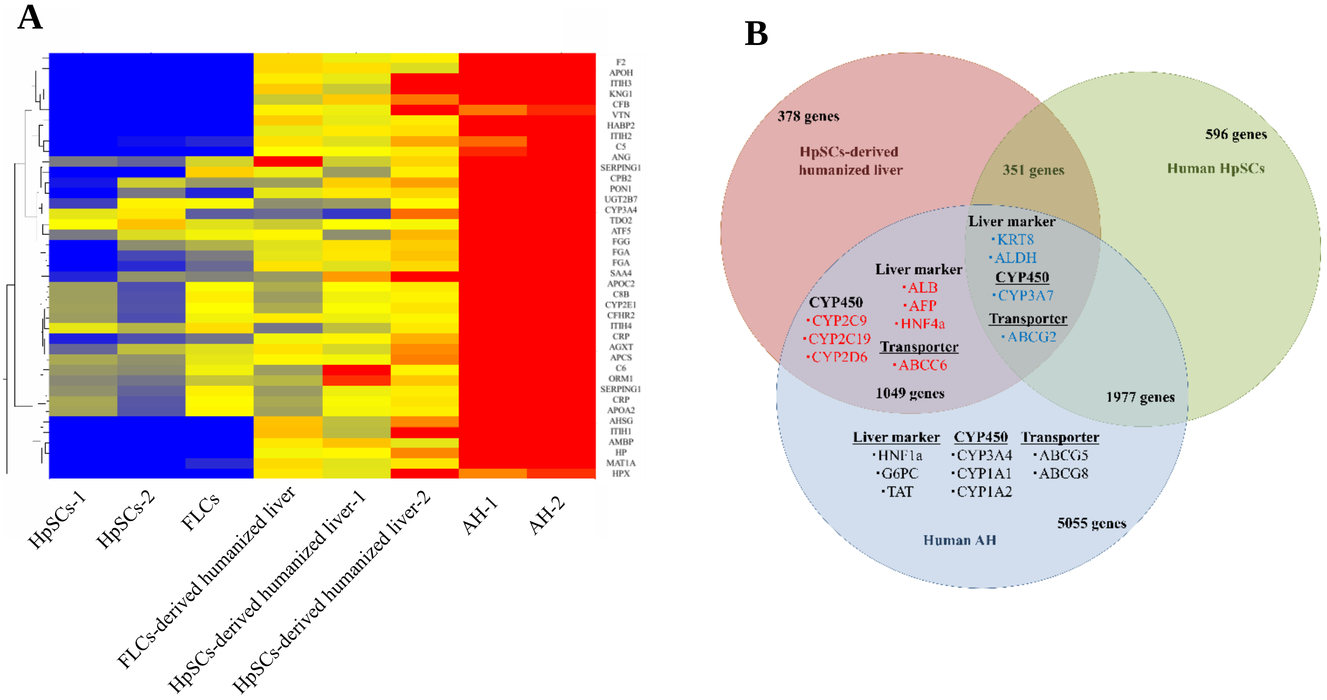

C

Genes	Human HpSCs	HpSCs-derived humanized liver	Human AH
**Phase I** (detoxification enzyme)	28.3%(15/53)	50.9%(27/53)	92.5%(49/53)
**Phase II** (liver signature factor)	61.6%(61/99)	86.8%(86/99)	96.9%(96/99)
**Phase III** (transporter)	66.6%(34/51)	68.6%(35/51)	94.1%(48/51)
**Total**	54.2%(110/203)	72.9%(148/203)	95.1%(193/203)

Figure S6. Liver specific genes and drug metabolism genes' expression in humanized livers.

A. Heat map for 38 liver specific signature genes, shown separately for independent experiments to analyze human primary fetal liver cells (FLCs) and hepatic stem cells

(HpSCs) before and at 8 weeks after cell transplantation. B. Venn diagram for the selective display of genes with high or lower expression in humanized livers derived from human HpSCs (2-fold changes): human HpSCs-derived humanized liver at 8 weeks after cell transplantation (upper left diagram); human AH (lower diagram); human HpSCs (upper right diagram). Overlapping regions show the gene numbers and representative gene names that were common in the different comparisons. (see Additional file 2 for the gene list in each comparison). C. Percentages of human drug metabolic phase I, II, and III genes that were positively detected in human HpSCs-derived humanized liver at 8 weeks after cell transplantation, human HpSCs, and human AHs. The numbers of genes detected and total gene numbers for each phase are indicated in brackets. (see Additional file 3 for the gene list). Human adult hepatocytes were used as a positive control.
